# The memory for time and space differentially engages the proximal and distal parts of the hippocampal subfields CA1 and CA3

**DOI:** 10.1371/journal.pbio.2006100

**Published:** 2018-08-28

**Authors:** Zachery Beer, Peter Vavra, Erika Atucha, Katja Rentzing, Hans-Jochen Heinze, Magdalena M. Sauvage

**Affiliations:** 1 Mercator Research Group, Functional Architecture of Memory Unit, Ruhr-University Bochum, Germany; 2 Otto von Guericke University, Medical Faculty, Functional Neuroplasticity Department, Magdeburg, Germany; 3 Leibniz-Institute for Neurobiology, Functional Architecture of Memory Department, Center for Learning and Memory, Magdeburg, Germany; 4 Otto von Guericke University, Neurology Department, Magdeburg, Germany; 5 Otto von Guericke University, Center for Behavioural Brain Sciences, Magdeburg, Germany; Institute of Science and Technology Austria, Austria

## Abstract

A well-accepted model of episodic memory involves the processing of spatial and non-spatial information by segregated pathways and their association within the hippocampus. However, these pathways project to distinct proximodistal levels of the hippocampus. Moreover, spatial and non-spatial subnetworks segregated along this axis have been recently described using memory tasks with either a spatial or a non-spatial salient dimension. Here, we tested whether the concept of segregated subnetworks and the traditional model are reconcilable by studying whether activity within CA1 and CA3 remains segregated when both dimensions are salient, as is the case for episodes. Simultaneously, we investigated whether temporal or spatial information bound to objects recruits similar subnetworks as items or locations per se, respectively. To do so, we studied the correlations between brain activity and spatial and/or temporal discrimination ratios in proximal and distal CA1 and CA3 by detecting *Arc* RNA in mice. We report a robust proximodistal segregation in CA1 for temporal information processing and in both CA1 and CA3 for spatial information processing. Our results suggest that the traditional model of episodic memory and the concept of segregated networks are reconcilable, to a large extent and put forward distal CA1 as a possible “home” location for time cells.

## Author summary

Departing from the most influential model of episodic memory (the two-streams hypothesis), we have recently proposed a new concept of information processing in the hippocampus according to which “what” one remembers and “where” it happens might be processed by distinct subnetworks segregated along the proximodistal axis of the hippocampus, a brain region tied to memory function, instead of being systematically integrated at this level. Here, we focused on the processing of temporal and/or spatial information in the proximal and distal parts of CA1 and CA3 in mice to test whether the two concepts are reconcilable. To do so, we used an imaging method with cellular resolution based on the detection of the RNA of the Immediate Early Gene (IEG) *Arc*, which is tied to synaptic plasticity and memory demands, and correlated imaging results with memory performance. Our data confirm the existence of subnetworks segregated along the proximodistal axis of CA1 and CA3 that preferentially process spatial and non-spatial information and suggest a key involvement of distal CA1 in temporal information processing. In addition, they show that the two models are complementary to a large extent and posit the “segregated” model as a viable alternative for the two-streams hypothesis.

## Introduction

**Fig 1:**
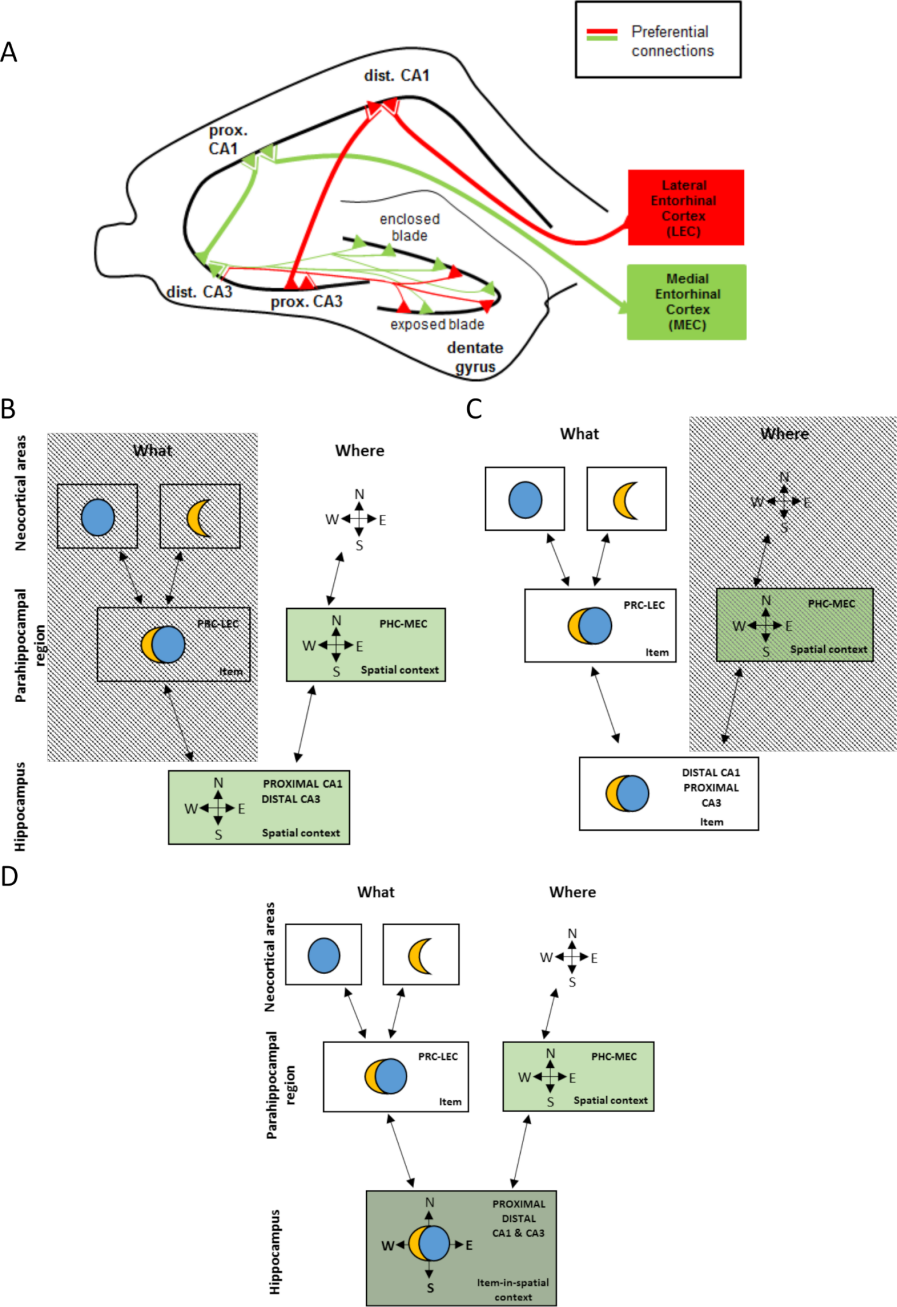
Schematic representation of the “spatial” (green) and the “non-spatial” (red) hippocampal subnetworks and conceptual schemas of models of spatial and non-spatial information processing in the medial temporal lobe. (A) The LEC and MEC project to CA1 at different proximodistal levels. Proximal CA3 projects preferentially to distal CA1, and distal CA3 projects preferentially to proximal CA1. Proximal CA3 is less connected to the enclosed blade of the DG than distal CA3. Of note, this diagram is not quantitative. For the sake of clarity, projections were drawn as reaching distinct neurons. However, the model does not imply this to be mandatory. (B–D) Schemas of models of information processing in the medial temporal lobe. According to the “segregated” view of information processing in the hippocampus [24,25], only the “spatial” subnetwork (proximal CA1 and distal CA3) would be recruited if the salient dimension of the memory was spatial (panel B), while only the “non-spatial” subnetwork (distal CA1 and proximal CA3) would be engaged if the salient dimension was non-spatial (panel C).Within the frame of the “two-streams model” [1,2], the spatial and non-spatial dimensions of an episode are integrated at the level of the hippocampus. In other words, both the “spatial” (i.e., distal CA3–proximal CA1) and the “non-spatial” (i.e., proximal CA3–distal CA1) hippocampal subnetworks would be recruited in this case (panel D). DG, dentate gyrus; dist., distal; LEC, lateral entorhinal cortex; MEC, medial entorhinal cortex; prox., proximal.

In the early 1980s, Mishkin and colleagues proposed a very influential model of episodic memory according to which spatial and non-spatial information emerging from the dorsal and the ventral visual pathways would be integrated into episodes at the level of the hippocampus [1,2]. This model considers information related to the features of objects and their location as non-spatial and spatial information, respectively, while the temporal information bound to these objects is not considered even though this dimension constitutes a key feature for the memory of episodes [3]. Clear empirical evidence for the integration of this spatial and non-spatial information at the level of the hippocampus and possible mechanisms underlying such an integration are still missing. In addition, it is not known whether such an integration would still take place if only one of the dimensions of the memory is salient, i.e., when the integration of both dimensions is not “necessary.” Moreover, the cortical areas constituting the last relay of the “extended” ventral and dorsal pathways, namely the lateral entorhinal cortex (LEC) and the medial entorhinal cortex (MEC), respectively, preferentially project at distinct proximodistal levels of the hippocampal subfield CA1. Indeed, the LEC that essentially processes non-spatial information preferentially projects to the distal part of CA1 (away from the dentate gyrus [DG], i.e., close to the subiculum; Fig 1A). In contrast, the MEC, more sensitive to spatial content, preferentially projects to the proximal part of CA1 (close to the DG and CA2) [4–13]. In addition, the proximal and distal parts of CA3 send segregated projections to CA1. Distal CA1 primarily receives projections from the proximal part of CA3 (close to the DG) and proximal CA1 from distal CA3 (away from the DG, i.e., close to CA2) [14–18]. Furthermore, the distal part of CA3 receives projections from the enclosed blade of the DG, which is tuned to spatial information, as well as from the crest and the exposed blade. Moreover, entorhinal cortex (EC) cells send most of their inputs at this level because EC cells synapse at the level of the lacunosum moleculare, which is quasi nonexistent at the proximal levels of CA3. In comparison, the proximal part of CA3 receives fewer projections from the enclosed blade of the DG and fewer entorhinal inputs, among which LEC inputs which preferentially deal with non-spatial content [19–23].

Altogether, these findings led us to recently suggest the existence of distinct “spatial” and “non-spatial” hippocampal subnetworks segregated along the proximodistal axis of the hippocampus that would preferentially be engaged either when only the spatial dimension or only the non-spatial dimension of a memory is salient, i.e., when the integration of both dimensions is not “necessary” (Fig 1B and 1C) [24,25]. These networks were termed “spatial” and “non-spatial” subnetworks with regards to their relative ability (i.e., not absolute) to process non-spatial and spatial information, i.e., the “non-spatial” subnetwork processes non-spatial information over spatial information, whereas the “spatial” network favors the processing of spatial information over non-spatial information.

Evidence for a functional segregation along the proximodistal axis of CA1 and CA3 is sparse, but its existence is supported by recent electrophysiological and *Arc* imaging studies. An electrophysiological study from the Moser laboratory reported a stronger engagement of proximal CA1 over distal CA1 for the processing of spatial stimuli [10]. Conversely, we showed a stronger recruitment of distal CA1 over proximal CA1 for the processing of non-spatial (odor-based) information in a previous *Arc* imaging study [24]. In addition, activity differences along the proximodistal axis of CA1 were reported to be attenuated with aging and proximodistal theta activity coherence to be reduced in the dorsal hippocampus of a rodent animal model of epilepsy [26–27]. Furthermore, some of these reports and others also showed a preferential involvement of proximal CA3 for the retrieval of non-spatial memory and that of distal CA3 for the processing of spatial locations [24–25]. Finally, a proximodistal segregation of CA3 was also reported in terms of pattern completion and pattern separation [28–29].

Still, very little is known about these hippocampal subnetworks. Specifically, it is unclear whether non-spatial information other than objects or odors, such as temporal information, would also preferentially recruit the proximal CA3–distal CA1 “non-spatial” network. This hypothesis is substantiated by the fact that studies focusing on temporal bridging have indeed targeted distal CA1/the distal half of CA1 [30–38]. However, their focus was not the investigation of proximodistal differences in CA1; therefore, whether temporal information also preferentially engages the “non-spatial” subnetwork remains to be thoroughly tested. Also, it is not known whether spatial information related to items such as objects (i.e., object-in-place information) would primarily engage the distal CA3–proximal CA1 “spatial” network, as is the case for locations. Finally, it is not clear whether the concept of segregated information processing in the hippocampus and the traditional model of episodic memory are supported by distinct neural substrates or whether they are variations of one and the same principle supported by the same neural networks that are recruited depending on the nature of the salient dimensions of the memory (Fig 1B–1D).

**Fig 2:**
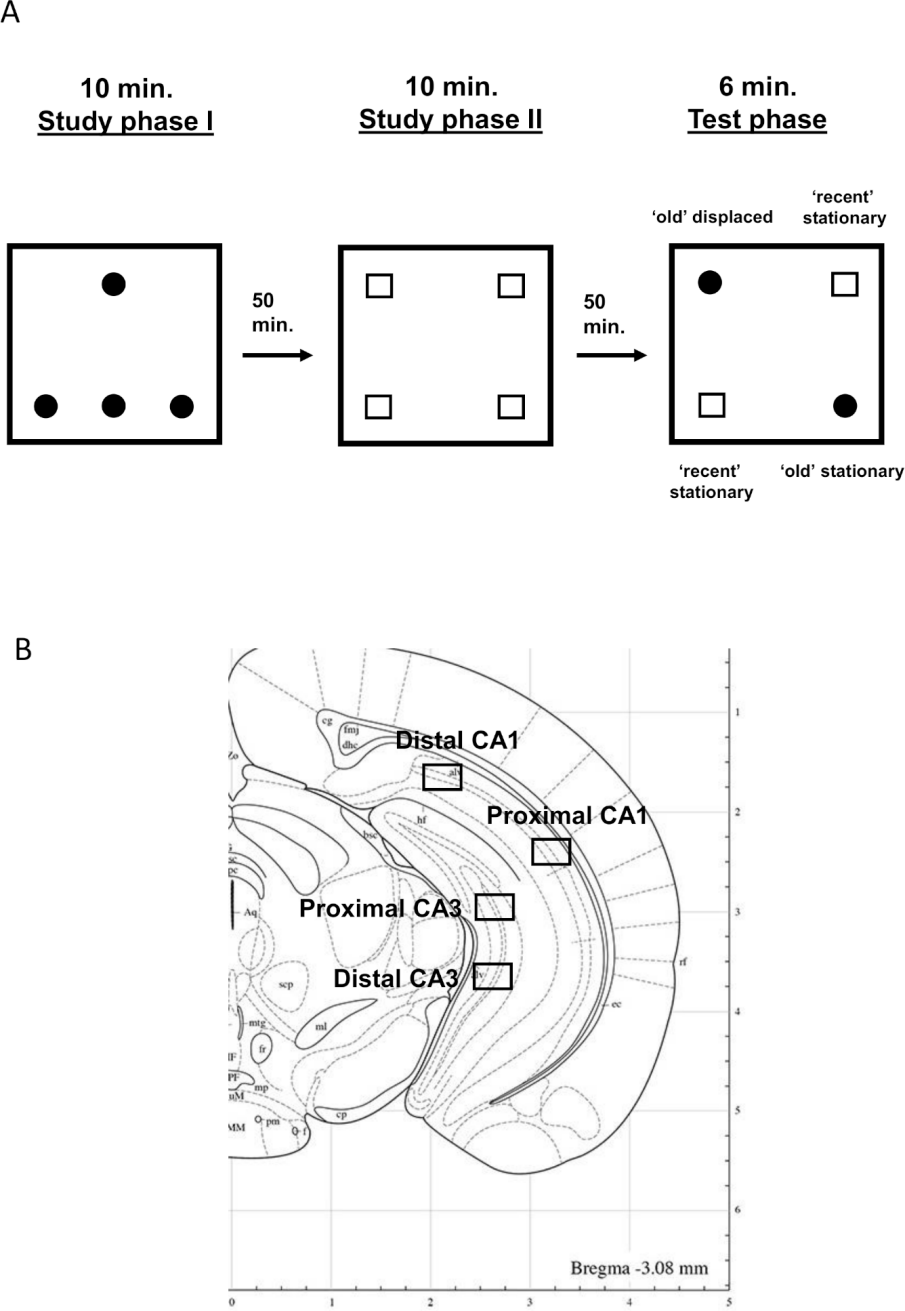
Memory task and location of the imaging frames. (A) Behavioral protocol. The animals were placed into an open field with 4 identical objects during the 10-min study phase 1, followed by a 50-min delay phase. After the first delay phase, animals returned to the same open field with a new set of 4 identical objects during the 10-min study phase 2. Following the second 50-min delay phase, memory for the spatial arrangement of the objects (where the objects were originally placed) and the temporal aspect of the task (when the objects were presented) was assessed by returning animals to the open field for a third time, with 2 copies of the objects from study phase 2 (recent stationary objects) and 2 copies of the objects from study phase 1 (“old” objects: one stationary and one displaced), and by measuring the time mice explored each object that was used to calculate discrimination ratios during the test phase. (B) Location of the imaging frames [49]. Black frames define the level at which images were taken with a 40× objective. Three images were taken per target area on nonconsecutive sections that covered approximately 400 microns. Counting was performed only on neurons as described in [43] and totaled approximately 270 neurons per area.

To address these questions, we investigated which areas—among the distal and proximal parts of CA1 and CA3—are tuned to temporal information, spatial information, or both types of information. To do so, we used a spontaneous object-recognition memory task that allows for the evaluation of distinct discrimination ratios for the retrieval of the temporal aspect of a memory (“when” the objects were presented) and that of its spatial aspect (“where” the objects were located) [39–42], Fig 2A).

**Fig 3:**
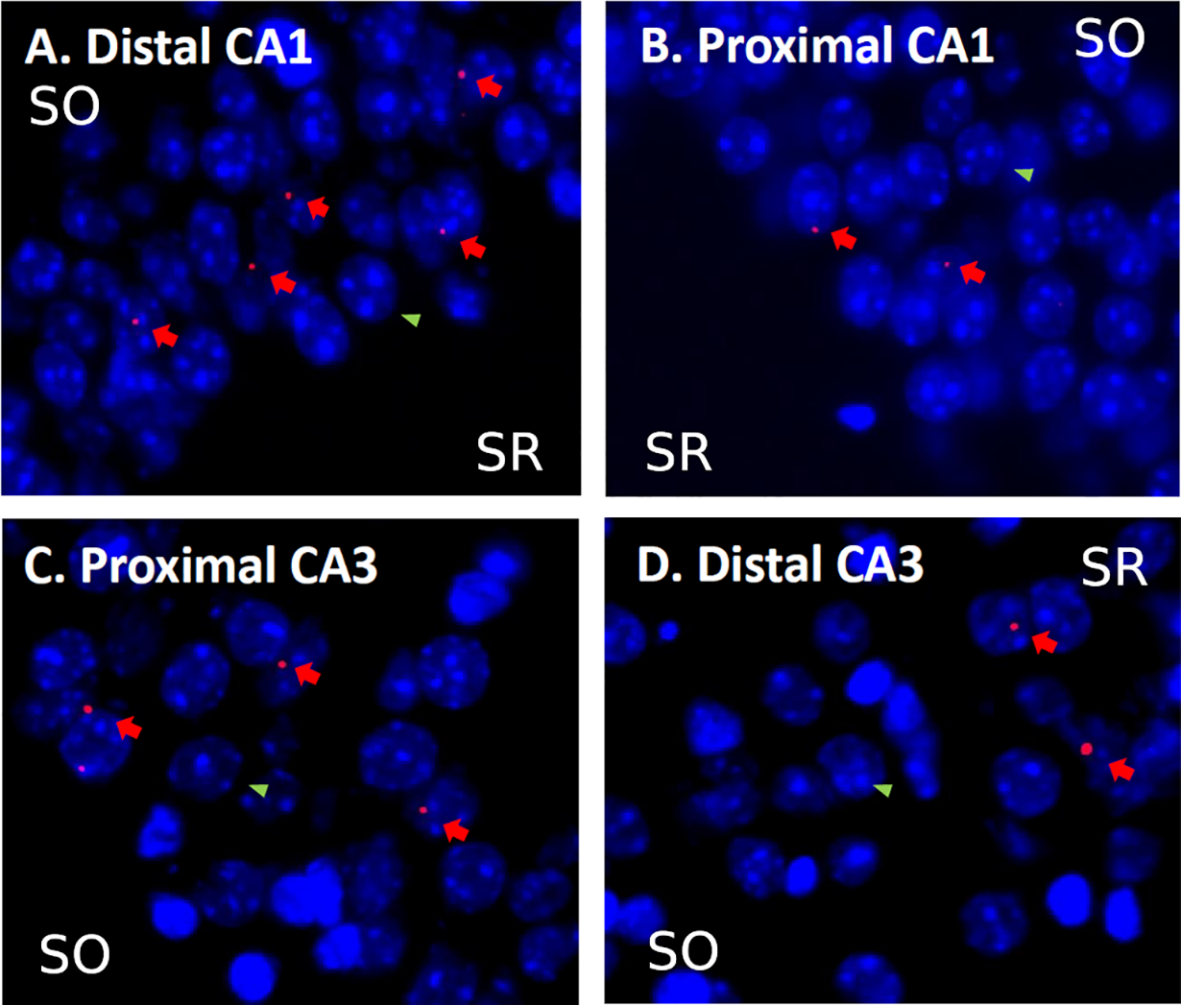
Examples of *Arc* labeling in CA1 and CA3. DAPI-stained neuronal nuclei are shown in blue. *Arc* intranuclear labeling in red. Red arrows show examples of *Arc*-positive cells, and green arrowheads show examples of *Arc-*negative cells. Example of *Arc* expression in (A) distal CA1, (B) proximal CA1, (C) proximal CA3, and (D) distal CA3. Scale bars: 20 μm. SO, stratum oriens [44]; SR, stratum radiatum [49].

Since performing electrophysiological recording simultaneously in 4 brain areas remains a major challenge and since the coordinates of the proximal and distal parts of CA1 and CA3 vary greatly along the transverse axis of the hippocampus because of its folding, we favored a high-resolution imaging approach (i.e., to the cellular level) over lesion/inactivation/optogenetic approaches because the latter approaches would be unlikely to yield the spatial resolution necessary to tease apart the specific function of the proximal and distal parts of CA1 and CA3 in mice. This molecular imaging technique is based on the detection of the RNA of the Immediate Early Gene (IEG) *Arc* that is commonly used to map brain activity in the medial temporal lobe [45–47] and is tightly linked to plasticity processes [48]. In addition, *Arc* is more sensitive to memory demands than other IEGs [24,49,50] and allows for each cell activated at test to be detected. *Arc* RNA is visualized with the help of fluorescent tags, which allow for the percentage of cells engaged at test to be evaluated (Fig 3A–3D).

## Materials and methods

### Ethics statement

Procedures were approved by the Ruhr Universität Bochum Institutional Animal Use Committee and the LANUV (8.87–51.04.20.09.323).

### Subjects

Adult male C57BL/6 mice (*n* = 26)—single-caged and kept under a reversed light/dark cycle—were tested during their active phase.

### Materials

The apparatus was a 32 × 32 × 41 cm open field, placed in a dimly lit room. Extra-maze cues, as well as cues on the outside wall of the open field, served as spatial references. A video camera (Sony, HDR/CX500E) recorded the animals’ behavior for off-line analysis. Six copies of 2 different metallic objects were used for testing so that objects used during the test phase were duplicates of those during the study phases. Pilot studies showed that animals could distinguish between the 2 objects and had no aversion or preference for either object. The location of the recent, old, stationary, and displaced objects was counterbalanced between animals.

### Habituation and testing

The habituation procedure occurred on 4 consecutive days, following a procedure described in Dere and colleagues [39]. In short, animals were habituated to the empty open field for 20 min during days 1 and 2. To encourage mice to explore all areas of the box (divided in 9 quadrants) and minimize the development of a spatial bias, 1 chocolate sprinkle was placed in the center of each quadrant. The absence or displacement of sprinkles and/or the presence of droppings in each quadrant at the end of each 20-min session were assessed and revealed that mice had explored each quadrant during each session. On days 3 and 4, animals were habituated to the presence of objects, which were not used on the testing day in conditions that mirrored those of the testing day (three 10-min trials, two 50-min delay periods). Animals were tested in groups of 9, plus 1 home-caged control that was placed in the same room but did not perform the task. On day 5, the testing procedure followed that of days 3 and 4, but the test phase was adapted for an optimal detection of *Arc* pre-mRNA (6-min test phase; Fig 2A). During study phase 1 and 2, animals were exposed to a set of 4 identical objects. During the first study phase, objects formed a triangle. During the second study phase, a different set of 4 identical objects formed a square. At test, duplicates of the objects previously studied were used. Two of the objects that had been the most recently explored (i.e., explored during study phase 2) were placed at the location they occupied then (the “recent stationary” objects). In addition, one of the objects that had been experienced earlier (i.e., during study phase 1) was also placed at the location it occupied then (the “old stationary” object), while another object of the same set was placed in a novel location (the “old displaced” object). After each trial, the open field and stimuli were cleaned with water and a solution containing 10% ethanol.

### Behavioral analysis

Based on animals’ natural preference for novelty [51], a successful memory for a given spatial location (e.g., a successful spatial discrimination) is observed when the “displaced old” object is explored more than the “stationary old” object, and a successful memory for the temporal context in which the object was experienced (e.g., a successful temporal discrimination) is reflected by a longer exploration of the “stationary” old object compared with that of the average of the 2 “recent” objects [39–42]. A response pattern according to which mice explore the old displaced object more than the old stationary object and concomitantly the old stationary object more than recent objects suggests that mice have the ability to establish an integrated memory for events comprising information about “what,” “where,” and “when” [39]. The exploration time for an object was defined as the time spent in exploring an object, i.e., directing the nose at a distance <2 cm to the object and/or touching it with the nose, as originally described in Ennaceur and Delacour (1988) [51]. The animals’ exploratory behavior was recorded during each phase for off-line analysis and was used to calculate spatial and temporal discrimination indices at test (spatial and temporal D2s, respectively).

Performance was scored manually by 2 independent experimenters blind to experimental conditions and averaged. The scores of both experimenters were highly correlated (r = 0.879). Specifically, D2 scores were calculated for each animal with the following equations: spatial D2 = (exploration time _displaced old object_ − exploration time _stationary old object_) ÷ total exploration time for both old objects; temporal D2 = (exploration time _stationary old object_ − average exploration time _both recent objects_) ÷ (exploration time _stationary old object_ + average exploration time _both recent objects_).

### Fluorescent in situ hybridization

Following the standard protocol for detection of *Arc*, animals were euthanized immediately after the test phase, and as *Arc* pre-mRNA was detected, only *Arc* intranuclear signal was observable [49,52–54]. In short, brains were removed, flash frozen in isopentane, and stored at −80°C until sectioning. Brains were sectioned with a cryostat (Leica CM 3050 S; 8-μm–thick coronal sections), mounted on Polylysine slides (Thermo Scientific), and stored at −80°C until in situ hybridization. *Arc* pre-mRNA probes were synthesized using the digoxigenin-labeled UTP kit (Roche Diagnostics). Following a similar fluorescent in situ hybridization (FISH) protocol as Nakamura and colleagues (2013) [24], slides were fixed with 4% buffered paraformaldehyde and rinsed with 0.1 M PBS. Slides were treated with an acetic anhydride/triethanolamine/hydrochloric acid mix, rinsed, and briefly soaked with a prehybridization buffer. The tissue was hybridized with the digoxigenin-labeled *Arc* probe overnight at +65°C. Following hybridization, slides were rinsed with buffer solutions and treated with an antidigoxigenin-horseradish peroxidase (HRP) conjugate (Roche Molecular Biochemicals) and a cyanin-5 substrate kit (CY5, TSA-Plus system, Perkin Elmer). Nuclei were counterstained with 4’, 6’-diamidino-2-phenylindole (DAPI; Vector Laboratories).

### Image acquisition and evaluation of *Arc* signal

To detect *Arc*, 1 slide per animal was processed. Slides contained 8 nonconsecutive brain sections (approximately −3.00 mm AP; Fig 2B) [44], and images from 3 nonadjacent sections distant approximately 200 microns (i.e., covering approximately 400 microns) at this AP level were acquired. The number of activated neurons was evaluated on approximately 90 neurons per image on 3 nonadjacent sections (i.e., on approximately 270 neurons per area of interest). Of note, the distal CA3 window is located in the central portion of CA3, and not more ventrally, because the very ventral portion of CA3 belongs to proximal CA3 (close to the DG). Images were captured with a Keyence Fluorescence microscope (BZ-9000E; Japan). Images were taken with a 40× objective (z-stacks of 0.7-μm–thick pictures; see example Fig 3A–3D). Exposure time and light intensity were kept similar for image acquisition. As first described in the seminal work of Guzowski and colleagues [49], contrasts were set to optimize the appearance of intranuclear foci [43, 44, 51, 52]. To account for stereological considerations, neurons were counted on 8-μm–thick sections that contained 1 layer of cells, and only cells containing whole nuclei were included in the analysis [55]. The quantification of *Arc* expression was performed in the median 60% of the stack in our analysis because this method minimizes the likelihood of taking into consideration partial nuclei and decreases the occurrence of false negative. This method is comparable to an optical dissector technique that reduces sampling errors linked to the inclusion of partial cells into the counts and stereological concerns because variations in cell volumes no longer affect sampling frequencies [56]. Also, as performed in a standard manner in *Arc* imaging studies, counting was performed on cells (>5 μm) thought to be pyramidal neurons or interneurons because small non-neuronal cells such as astrocytes or inhibitory neurons do not express *Arc* following behavioral stimulation [57]. The designation “intranuclear-foci–positive neurons” (*Arc*-positive neurons) was given when the DAPI-labeled nucleus of the presumptive neurons showed 1 or 2 characteristic intense intranuclear areas of fluorescence. DAPI-labeled nuclei that did not contain fluorescent intranuclear foci were counted as “negative” (*Arc*-negative neurons) [49]. Percentage of *Arc*-positive neurons was calculated as follows: *Arc-*positive neurons ÷ (*Arc*-positive neurons + *Arc-*negative neurons) × 100. The home-caged group was generated to control for *Arc* baseline expression, which is known to be low (S1 Fig and S4 Data).

### Statistical analysis

All statistical analyses were implemented in the R statistical package (version 3.4.2). To assess the relationship between *Arc* expression and the spatial and temporal discrimination indices D2_space_ and D2_time_, 2 complementary analyses were conducted that led to comparable results: (i) fitting a linear mixed model to the *Arc* expression using the continuous discrimination indices and (ii) standard correlation analyses for each region separately.

For the first analysis, we estimated a linear mixed model that is conceptually comparable to a linear regression or partial correlations but explicitly models the repeated measurement of *Arc* expression from the same animals in the 4 brain regions and thus allows a comparison of the estimated effects across brain regions, as well as a comparison of the effects of both discrimination indices directly [58]. We used the “mixed” function in the “afex” package (version 0.18) [59], which in turn uses the “lme4” package (version 1.1) [60] for the estimation; *p*-values were computed using the Satterthwaite approximation of the degrees of freedom when assessing the significance of the fixed effects as well as using parametric bootstrapping, as implemented by the “mixed” function. The linear mixed model consisted of fixed effects for categorical variables “region” (CA1 and CA3) and “proximodistal” (proximal and distal) and the two discrimination indices (D2_time_ and D2_space_) and their interactions. We specified a random intercept per animal as a random factor to explicitly model the repeated measurement of *Arc* expression. To conduct post hoc comparisons, we computed area-specific mean activity and the slopes of temporal and spatial discrimination indices of the fixed effects using the “lsmeans,” “lstrends,” and “cld functions” in the “lsmeans” package (version 2.27) [61]. Whether these area-specific effects were significantly different from 0 was assessed by inspecting the 95% CI—if the interval does not include 0, the effect is considered significantly different from 0.

By modeling the influence of the increase in both discrimination indices concurrently, we also estimated the increase in *Arc* expression when both the spatial and temporal discriminations were successful. Specifically, the slope for “space+time”—D2_space+time_—which quantifies this increase, was estimated by calculating the sum of the slopes of spatial and temporal discrimination indices.

For visualization purposes, contour lines were used to represent the relative relationship between the discrimination indices and the *Arc* activity as predicted by the fitted model. Contour lines are extrapolated beyond experimental data points by visualizing the model prediction at those hypothetical discrimination ratios. Each line represents the set of spatial and temporal discrimination indices for which the mixed model predicts the same level of *Arc* activity. Note that these lines are parallel because the underlying model assumes a linear combination of the discrimination indices D2_time_ and D2_space_. A non-linear model including quadratic terms and the interaction of the 2 discrimination indices gave comparable results, therefore only the simpler linear model is reported here.

For the second analysis (ii), we calculated standard correlation coefficients between the *Arc* expression and the spatial and temporal discrimination indices, respectively. This straightforward analysis minimizes potential problems related to misspecifying the above mixed model that could lead to higher rates of false positives [62] but does not explicitly model the influence of both discrimination indices at the same time (i.e., correlations between *Arc* expression and the spatial D2 ignore the potential influence of temporal D2, and vice versa).

## Results

### Temporal and spatial discrimination ratios

**Fig 4:**
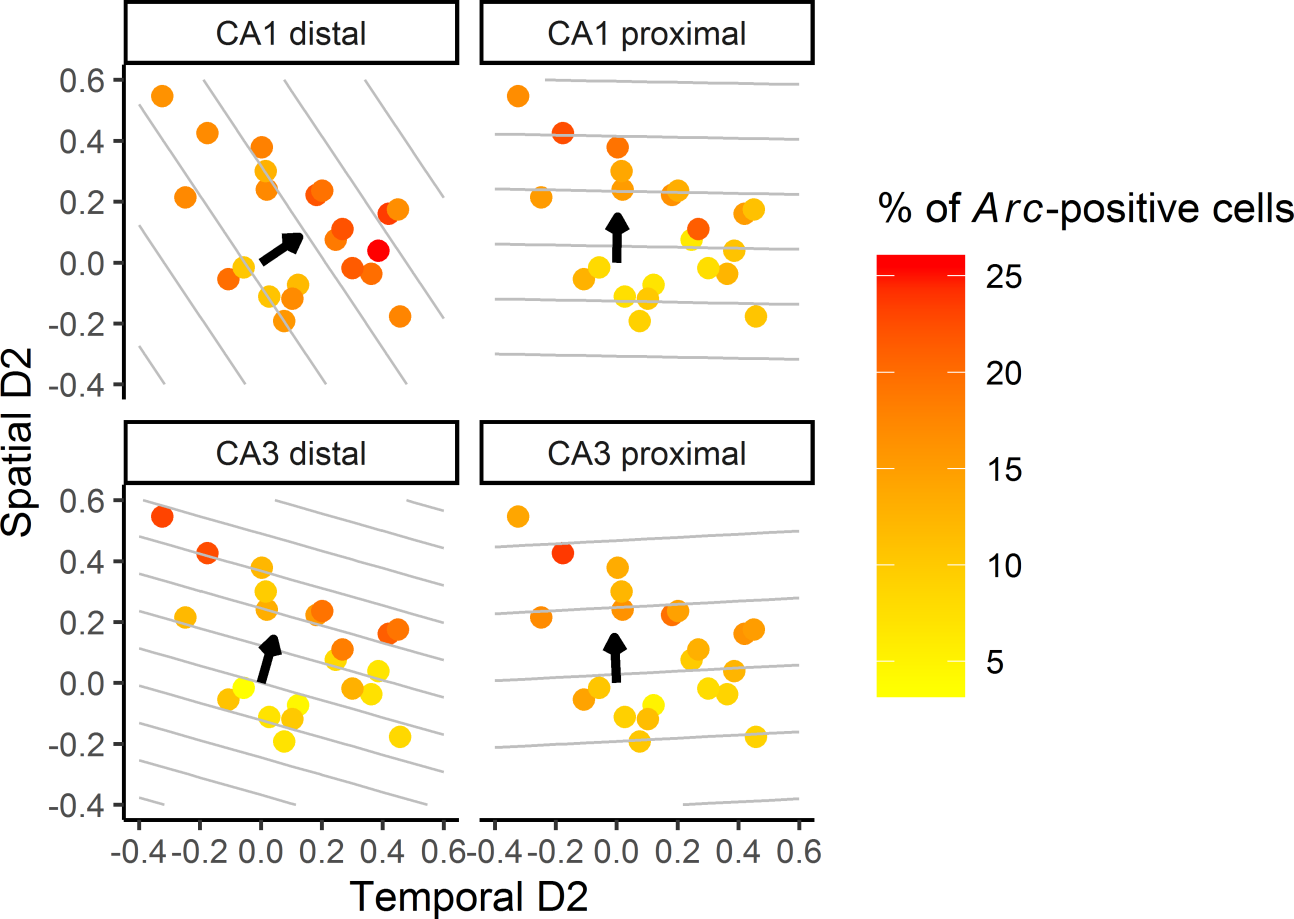
*Arc* expression varies with temporal and spatial discrimination in a distinct manner in the distal and proximal parts of CA1 and CA3. Scatter plots of *Arc* expression as a function of both temporal and spatial D2 ratios. Overlaid are contour lines predicted by the linear mixed model that estimates each area’s level of *Arc* activity based on both D2 ratios concurrently. Distal CA1 shows the strongest increase in activity with increasing retrieval of temporary information (i.e., contour lines are more vertical), while *Arc* expression increases more with retrieval of spatial information in all other areas (i.e., contour lines are more horizontal). The arrow indicates the preferred direction of *Arc* variation (underlying data in Supporting Information S2 and S3 Data).

Patterns of object exploration varied between animals, leading to a substantial spread of the temporal and spatial discrimination indices (Fig 4). Discrimination indices did not correlate with the total objects exploration time during study phases 1 and 2 (66.48 s ± 6.19 s [D2_time_: r = 0.21; *p* = 0.34; and D2_space_: r = 0.056; *p* = 0.806] and 66.26 s ± 7.49 s [D2_time_: r = −0.07; *p* = 0.75; and D2_space_: r = −0.015; *p* = 0.95], respectively]) nor at testing (48.86 s ± 4.05 s [D2_time_: *r* = 0.17; *p =* 0.46; and D2_space_: *r* = 0.11; *p =* 0.64]; and see also S1 Table and S1 Data), indicating that differences in total object exploration time per se could not account for the differences in discrimination indices reported in the present study.

To assess the relationship between *Arc* expression and the spatial and temporal discrimination indices, D2_space_ and D2_time_, the following 2 complementary analyses were conducted: (1) fitting a linear mixed model to the *Arc* expression using the continuous discrimination indices and (2) performing standard correlation analyses between *Arc* expression and the discrimination indices.

### Distal CA1 is especially sensitive to temporal information, whereas proximal CA1 and CA3 and distal CA3 are primarily tuned to spatial information

A contour plot of predicted *Arc* expression as a function of the spatial and temporal D2s (Fig 4) showed that, while the recruitment of all 4 areas varied with the spatial D2 (albeit to different degrees), distal CA1’s engagement varied to a larger extent with the temporal D2, as indicated by the contour lines for distal CA1 being more vertical than horizontal. In contrast, the contour lines for proximal CA1 and CA3 and distal CA3 (being more horizontal than vertical) reflected a stronger sensitivity for spatial discrimination.

In addition, statistical comparisons of the slope of D2_time_ showed that *Arc* expression varied with the temporal D2—especially in distal CA1 (b = 11.32) and to a lesser extent in distal CA3 (b = 6.95)—but failed to do so in other areas (proximodistal by D2_time_ interaction: χ^2^(1) = 11.13; *p* = 0.002; all other effects: all *p* > 0.12) (see also S1A and S1B Table). Moreover, further post hoc comparisons revealed that activity levels increased more as the temporal discrimination became higher in distal CA1 than in proximal CA1 (b = 0.30) or CA3 (b = 3.67) (both *p* < 0.005) and that distal CA3 activity varied more with D2_time_ than proximal CA3 activity (*p* = 0.048). Notably, investigation of the CIs of the slopes underlined the robustness of the findings for distal CA1, as the standard 95% CI of the slope of D2_time_ excluded 0 only in distal CA1 (i.e., the slope differed from 0), while a more relaxed 90% CI was necessary to get similar results for distal CA3. In other words, the slope of D2_time_ differed from 0 for distal CA1 but not for distal CA3, suggesting a more robust tuning of distal CA1 than distal CA3 to temporal information. In summary, under standard statistical criteria, the present results suggest that especially distal CA1 is sensitive to the retrieval of temporal information.

In contrast to the D2_time_ slopes, comparisons of the spatial D2 slopes showed that *Arc* expression increased in all areas as a function of spatial discrimination, although the extent to which this was the case differed by area (D2_space_ effect: χ^2^(1) = 17.98; *p* = 0.001; region by D2_space_ interaction: χ^2^(1) = 5.20; *p* = 0.030; region by proximodistal by D2_space_ interaction: χ^2^(1) = 10.16; *p* = 0.002; no other interaction effect: *p* > 0.050; see also S1 Table). Indeed, post hoc comparisons showed that *Arc* expression in distal CA1 (b = 7.56) increased the least with increasing spatial discrimination and that this increase was not significantly different from 0 (i.e., the standard 95% CI for the slope of D2 space included 0, but a less strict 90% CI did not). In addition, the D2_space_ slopes for the areas part of the “spatial” subnetwork—distal CA3 (b = 24.52) and proximal CA1 (b = 16.62—were larger than those part of the “non-spatial” subnetwork—distal CA1 (b = 7.56) and proximal CA3 (b = 13.49) (distal CA3 versus proximal CA3: *p* = 0.013; distal CA3 versus distal CA1: *p* = 0.0002; proximal CA1 versus distal CA1: *p* = 0.037). Thus, these results indicate that proximal CA1 and CA3, and distal CA3, are most tuned to spatial information, whereas distal CA1 is least tuned to spatial information.

Finally, estimating the influence of both discriminations simultaneously (i.e., predicting how *Arc* activity changes when both dimensions are recalled as captured by the slope of “D2_space+time_”) revealed that all areas were recruited at a comparable level (main effect of D2_space+time_: F_(1,19)_ = 15.41; *p* = 0.0009; no other significant effects, all *p* > 0.05; see S1C Table).

Altogether, the linear mixed-model approach indicates that *Arc* activity in the distal CA1 most strongly relates to retrieving temporal information and that proximal CA1 and CA3 and distal CA3 are especially tuned to spatial information.

### Standard correlation analyses confirm a preferential tuning of distal CA1 to temporal information, and a preferential tuning of all other areas to spatial information

**Fig 5:**
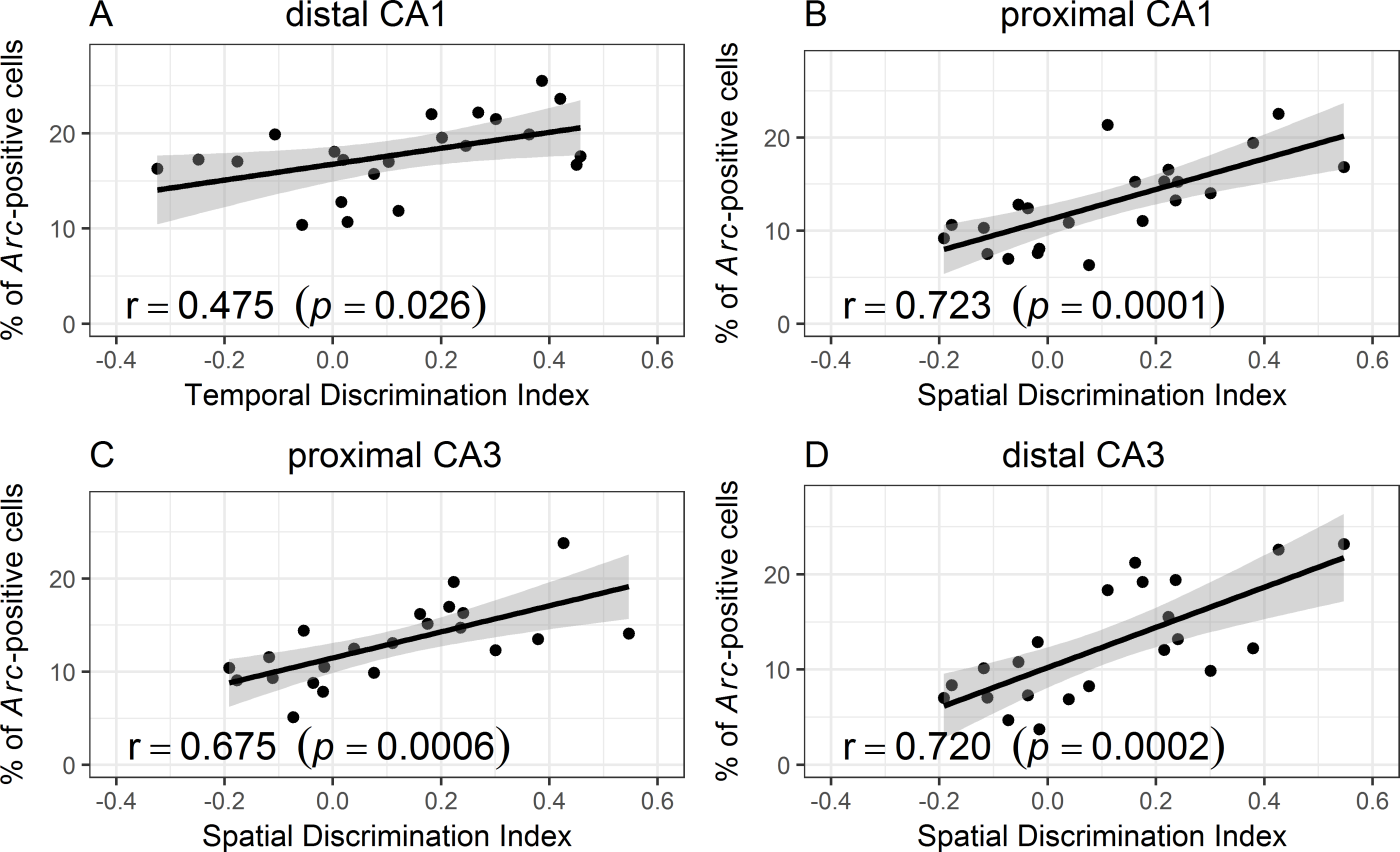
*Arc* expression correlates with memory performance (D2 ratios). *Arc* expression in (A) distal CA1—the area hypothesized to primarily process temporal information—strongly correlates with the temporal D2 scores, whereas no other hippocampal region does. In contrast, (B) proximal CA1 and (C and D) both parts of CA3—candidate areas for spatial information processing—correlate with the spatial discrimination index (spatial D2), whereas distal CA1 does not (S2 Fig for nonsignificant correlations; underlying data in Supporting Information S2 and S3 Data).

As observed with the linear mixed-model approach, distal CA1 correlated with the temporal discrimination index (r = 0.475; *p* = 0.026; Fig 5) but not with the spatial discrimination index (*p* = 0.65; S2 Fig). In contrast, all other areas correlated with the spatial discrimination index (proximal CA1: r = 0.723; *p* < 0.0001; proximal CA3: r = 0.675; *p* = 0.0006; distal CA3: r = 0.720; *p* = 0.0002; Fig 5) but not with the temporal discrimination index (proximal CA1: *p* = 0.17; proximal CA3: *p* = 0.14; distal CA3: *p* = 0.66; S2 Fig).

In summary, these analyses show, in line with the mixed-modeling results, that distal CA1 is especially tuned to temporal information and proximal CA1 and CA3 as well as distal CA3 are tuned to spatial information.

## Discussion

In the present study, we showed that processing temporal information bound to an object engages distal CA1 over proximal CA1, while processing spatial information about this object recruits proximal CA1 over distal CA1. In a striking contrast, distal CA3 was only slightly activated for the recall of temporal information, and both parts of CA3 were tuned to spatial information processing, albeit distal CA3 to a larger extent than proximal CA3. In addition, retrieving both types of information leads to a strong and comparable recruitment of all areas. Thus, our results suggest that retrieving one or more dimensions of a memory might rely on the same mechanism(s), supporting both the “segregated” and the “integrative” views of information processing in the hippocampus.

### The proximodistal functional segregation is more pronounced in CA1 than CA3

One question we addressed in this study is whether the temporal content of an event is preferentially processed by the proximal CA3–distal CA1 “non-spatial” subnetwork as it was the case for other non-spatial information, such as odors [24]. This appears to be the case at the level of distal CA1 because *Arc* expression increased in this area as the temporal discrimination ratio did (Fig 4). In addition, the slope of temporal D2 was higher in distal CA1 than in proximal CA1 (or any other areas), showing for the first time that the temporal context of an event is topographically organized along the proximodistal axis of CA1. These results also indicate that processing temporal information recruits the same part of the “non-spatial” subnetwork as other non-spatial information, such as odors [24]. Moreover, *Arc* expression in distal CA1 correlates only with temporal discrimination ratios (and not with spatial ratios; S2 Fig), further supporting the idea of a selective role of distal CA1 in the processing of temporal information. The recruitment of distal CA1 is unlikely to solely reflect the processing of object information as activity patterns are strikingly different between areas despite the fact that the same objects’ information (in time or space) is processed.

In contrast to CA1, CA3 was not engaged to a critical extent in processing temporal information, indicating that the temporal content is differentially computed than object or odor information at this level. These findings provide further support to the lesion, in vivo electrophysiology, and optogenetic studies that have indicated a preferential role of CA1 in temporal information processing over that of CA3 and suggest that distal CA1 is likely to be the home location of the “time cells” recently identified in CA1 [31–38,63,64]. As a support for the latter hypothesis, a thorough review of these studies showed that distal CA1/the distal half of CA1 was indeed targeted in these reports. The hypothesis of a preferential involvement of distal CA1 in the processing of time is also indirectly supported by evidence from a trace eye-blinking conditioning study showing that reversible inactivation of the LEC (which preferentially projects to distal CA1) impairs the retrieval of a memory for an association between temporally discontiguous stimuli [65]. Likewise, in vivo electrophysiology and IEG studies showed that the perirhinal cortex, which provides major inputs to the LEC, plays an important role for temporal-order memory and for object memory across large delays [66–68]. This might indicate that the LEC is the source of temporal information provided to distal CA1. Preliminary data from the Moser laboratory using population-level analyses of electrohysiological recordings partly support this hypothesis by reporting that LEC’s involvement within this frame depends on tasks’ demands, with free foraging tasks eliciting a stronger temporal representation in the LEC than continuous alternation/back-and-forth running tasks [69]; of note, such tasks’ demand dependency in the LEC were also reported in recent lesion and *Arc* imaging studies, albeit for the processing object and space information [70,71]. Our findings of a preferential involvement of distal CA1 in time processing depart from the standard model of episodic memory which, by extrapolation, predicts that temporal information would rather be processed by proximal CA1 because it mainly receives projections from the MEC, a part of the “where–when” pathway [2,4]. Even though the effect of the reversible inactivation of the MEC on temporal encoding in CA1 might be controversial [65,72], the latter hypothesis is supported by some electrophysiology studies that brought evidence of an involvement of the MEC in the integration of elapsed time and distance and in the temporal organization of CA1 activity [73,74]. Thus, further studies comparing directly LEC and MEC function within this frame will be necessary to clarify the nature and the extent of the contribution of the LEC and the MEC to temporal information processing and, by extension, the role of the distal and proximal parts of CA1 within this frame.

The second question of the present study was to assess whether the processing of spatial information bound to objects was topographically organized along the proximodistal axis of the hippocampus as it was shown for locations [25], i.e., whether it would also preferentially recruit the “spatial” hippocampal subnetwork. Our results show that, in addition to increasing with the temporal discrimination ratio, *Arc* expression in distal CA1 also increased as a function of the spatial discrimination index, indicating a relative tuning of distal CA1 to spatial information (Fig 4). However, and possibly as a further token of a preferential involvement of distal CA1 in the processing of temporal information, distal CA1 was the least tuned to the spatial discrimination when compared to all other areas. Indeed, the slopes of the spatial discrimination index for distal CA3 and proximal CA1 and CA3 were all larger than that of distal CA1. A key involvement of these regions was further supported by standard correlations and mixed-model analyses showing that *Arc* expression in proximal CA1 and both parts of CA3 correlated with spatial but not with temporal discrimination indices. This result confirms the central role of CA1 and CA3 in spatial memory as well as the existence of a functional segregation between the CA1 and CA3 subfields [30,75,76]. Moreover, because distal CA1 receives preferential projections from the LEC and proximal CA1 from the MEC, our findings are, by extension, in agreement with in vivo electrophysiology and Fos imaging studies that have shown that the LEC and the MEC are involved in the processing of object-in-place information [12,77–79]. These studies, however, did not directly assess the contribution of the proximal and distal parts of CA1 to the memory for object in place, but see Ito and Schuman [13]. In addition, our results show that processing spatial information bound to objects recruits the same part of the “spatial” subnetwork as processing locations because proximal CA1 was more tuned to spatial discrimination than distal CA1, and they indicate that the processing of object-in-place information is also topographically organized along the proximodistal axis of CA1. This finding, together with recent studies reporting a stronger engagement of proximal CA1 in the case of contextual changes and a weaker recruitment for non-spatial memory, shows that CA1’s functional segregation holds in various experimental settings and that the mechanism sustaining spatial information processing in CA1 could be the same when the information processed is related to a context or to an object (the object’s location) [24,10].

In CA3, a robust proximodistal segregation was also observed in terms of processing spatial information because distal CA3 was more tuned to spatial information than proximal CA3. This engagement of CA3 for spatial information processing is in line with previous lesion and electrophysiology studies—which, however, did not dissociate the contribution of proximal and distal parts of CA3 [80,81]. For example, lesions of CA3 impair object–place or odor–place–paired associations [82], and in vivo electrophysiological studies showed that spatial firing patterns in CA3 distinguish different environments in a foraging task [38]. Conversely, lesions of CA3 did not affect performance on an object–trace–odor task [63]. This stronger involvement of distal CA3 over proximal CA3 in dealing with object-in-place information matches results of a previous finding reporting a similar pattern for the processing of locations with a high-demand memory task [24], indicating that the proximodistal functional segregation in CA3 also holds independently of the type of spatial information processed (i.e., locations or object-in-place).

Thus, altogether, these data show that, at the exception of the temporal information in CA3, the retrieval of spatial (locations) or non-spatial (temporal) information bound to objects engages the same parts of CA1 and CA3 as retrieving information related to objects/odors or locations alone, respectively, indicative of a robust segregation of the spatial and non-spatial information along the proximodistal axis of CA1 and CA3.

### The “segregated” and “integrated” views of information processing in the hippocampus are reconcilable

Finally, in the present study, we also asked whether the concept of a segregated processing of spatial and non-spatial information in the hippocampus and the standard concept of an integration of this information at this level are “compatible” and based on the same networks. To be “compatible,” we hypothesized that, during memory retrieval, one of the subnetworks (“spatial” or “non-spatial”) would be recruited over the other when only one dimension of the memory is salient (spatial or non-spatial). In contrast, all areas would be activated to comparable levels when both dimensions are salient, i.e., no proximodistal differences would be observable in this case. Here, we report that, at the exception of the temporal information in CA3, proximodistal differences fitting the description of the “spatial” or “non-spatial” subnetworks were detected when animals discriminated on the basis of only spatial or temporal information as captured by the comparisons of the slopes of spatial or temporal discrimination indices, respectively. In addition, all areas were engaged to a similar extent when animals successfully discriminated based on the concurrent retrieval of both dimensions as substantiated by the comparisons of the slopes of the discrimination indices for space + time. Thus, these results indicate that the “segregated” and the “integrated” views of information processing in the hippocampus might be, to a large extent, based on the same principle(s) and networks but might differ in the nature and the number of dimensions of the memory to be retrieved.

### Cautionary notes

The present study focused on assessing the tuning of the proximal and distal parts of CA1 and CA3 to spatial and temporal information. Assessing whether the spatial and the non-spatial dimension of the memory are combined or kept segregated when both dimensions are retrieved, identifying the specific processes underlying the patterns of activity reported, or the basis of interindividual differences in behavioral performance (i.e., whether mice preferentially processed temporal and/or spatial information or failed to do so) are beyond the scope of the study and will require further investigations. Moreover, despite the fact that *Arc* expression was reported to better reflect behavioral task demands than other IEGs, such as *c-fos* and *zif268*, and not simply stress levels or motor activity [24,49,50], the latter processes and others might still partially contribute to the levels of *Arc* expression observed at test. For this reason, it was crucial to keep experimental conditions (handling, number of stimuli, locomotor activity, etc.) identical across animals. Since under this condition it could be ruled out that between-area differences could not stem from differences in total objects exploration times (comparable for phase 1, 2, and 3) or from neophobia (all objects were experienced prior to the testing phase), between-area comparisons of *Arc* expression are expected to reflect the processing of spatial and/or temporal information. Furthermore, the proximodistal differences in patterns of activity reported here are unlikely to be a by-product of the anatomical levels at which CA1 and CA3 are imaged because proximodistal differences at these levels were also reported independently of whether the septal level, the temporal level, or the transverse axis of the hippocampus (at which the proximal and the distal parts of CA1 and CA3 are located at different dorsoventral levels) were imaged in a previous study [24].

In summary, these findings complement our recent studies that revealed that spatial information (location) and non-spatial information (odors) can be processed in a segregated manner within the hippocampus [24,25] by showing that temporal and spatial information bound to objects engage, at least part of, the same subnetworks. In addition, we identified the distal part of CA1 as a potential “hub” for time cells and showed that the new concept of segregated processing of spatial and non-spatial information within the hippocampus is, to a large extent, reconcilable with the traditional view of an integration of this information at the level of the hippocampus.

## Supporting information

S1 Fig *Arc* expression for the “Home-cage” group (i.e., baseline *Arc* expression). As shown in previous studies, baseline *Arc* expression in home-caged mice, which did not perform the task but were present in the experimental room, was very low and ranged from 3.12% ± 0.86% to 3.4% ± 0.29%. This expression was comparable between proximal and distal parts of CA1 and CA3 (no significant region, proximodistal, or interaction effects). Bars represent group averages ± SEMs (underlying data in S4 Data).
(TIF)

S2 Fig Complement to Fig 5 (i.e., additional nonsignificant correlation plots). *Arc* expression in proximal CA1 and CA3 and in distal CA3 does not correlate with the temporal discrimination index nor does *Arc* expression correlate with the spatial discrimination index in distal CA1 (underlying data in S1–S3 Data).
(TIFF)

S1 Table Mixed-model analysis of Arc expression levels as function of discrimination indices. (A) ANOVA table of the mixed model: the model consists of fixed effects for the continuous discrimination indices and dichotomous “region” (CA1 versus CA3) and “proximodistal” (proximal versus distal) factors, as well as a random intercept for each animal. (B) Slopes for the spatial and temporal discrimination indices and post hoc comparisons of their difference. The slope of the trendline for temporal discrimination for distal CA1 is the steepest and the only one different from 0, indicating the highest correlation of *Arc* activity with temporal discrimination in this area (of note, D2_time_ by region by proximodistal interaction effect is not significant because b_time_ just failed to reach significance for distal CA3). In addition, distal CA1 activity increased the least with increasing spatial discrimination (b_space_ not different from 0), reflecting the weakest correlation with spatial discrimination for this area. (C) ANOVA table of mixed model of D2_space+time_, and interactions with “region” and “proximodistal” (underlying data in S1 Data, S2 Data and S3 Data).
(XLSX)

S1 Data Total object exploration times (s) per mouse for phases 1, 2, and test. (CSV)

S2 Data Individual exploration times (s) of each object at test (used to calculate discrimination ratios). (CSV)

S3 Data Individual *Arc* expression (expressed as percentage of *Arc-*positive neurons) in distal and proximal CA1 and CA3 subregions of the hippocampus at test (used to perform correlations). (CSV)

S4 Data Individual *Arc* expression (expressed as percentage of *Arc-*positive neurons) in distal and proximal CA1 and CA3 subregions of the hippocampus of home-caged control mice. These mice did not perform the task and served to confirm that baseline *Arc* expression is low (these values were used for S1 Fig).
(CSV)

## Abbreviations

DG: dentate gyrus
EC: entorhinal cortex
FISH: fluorescent in situ hybridization
HRP: horseradish peroxidase
IEG: Immediate Early Gene
LEC: lateral entorhinal cortex
MEC: medial entorhinal cortex
SO: stratum oriens
SR: stratum radiatum

## References

[1] Mishkin M, Ungerleider L. Contribution of striate inputs to the visuospatial functions of parieto-preoccipital cortex in monkeys. Behav Brain Res. 1982;6(1): 55–57. 10.1016/0166-4328(82)90081-x 7126325

[2] Mishkin M, Ungerleider L, Macko K. Object vision and spatial vision: two cortical pathways. Trends in Neurosci. 1983;6: 414–417.

[3] Tulving E. Precise of elements of episodic memory. Behav Brain Sci. 1984;7: 223–238.

[4] Tamamaki N, Nojyo Y. Preservation of topography in the connections between the subiculum, field CA1, and the entorhinal cortex in rats. J Comp Neurol. 1995;353(3): 379–390. 10.1002/cne.903530306 7538515

[5] Suzuki WA, Miller EK, Desimone R. Object and place memory in the macaque entorhinal cortex. J Neurophysiol. 1997;78(2): 1062–1081. 10.1152/jn.1997.78.2.1062 9307135

[6] Young BJ, Otto T, Fox GD, Eichenbaum H. Memory representation within the parahippocampal region. J Neurosci. 1997;17(13): 5183–5195. 9185556 10.1523/JNEUROSCI.17-13-05183.1997 PMC6573311

[7] Naber PA, Lopes da Silva FH, Witter MP. Reciprocal connections between the entorhinal cortex and hippocampal fields CA1 and the subiculum are in register with the projections from CA1 to the subiculum. Hippocampus. 2001;11(2): 99–104. 10.1002/hipo.1028 11345131

[8] Fyhn M, Molden S, Witter MP, Moser El, Moser MB. Spatial representation in the entorhinal cortex. Science. 2004;305(5688): 1258–1264. 10.1126/science.1099901 15333832

[9] Hargreaves EL, Rao G, Lee I, Knierim JJ. Major dissociation between medial and lateral entorhinal input to dorsal hippocampus. Science. 2005;308 (5729): 1792–1794. 10.1126/science.1110449 15961670

[10] Henriksen EJ, Colgin LL, Barnes CA, Witter MP, Moser MB, Moser El. Spatial representation along the proximodistal axis of CA1. Neuron. 2010;68(1): 127–137. 10.1016/j.neuron.2010.08.042 20920796 PMC3093538

[11] Burke SN, Maurer AP, Nematollahi S, Uprety AR, Wallace JL, Barnes CA. The influence of objects on place field expression and size in distal hippocampal CA1. Hippocampus. 2011;21(7): 783–801. 10.1002/hipo.20929 21365714 PMC3314262

[12] Deshmukh SS, Knierim JJ. Representation of non-spatial and spatial information in the lateral entorhinal cortex. Front Behav Neurosci. 2011;5: 69 10.3389/fnbeh.2011.00069 22065409 PMC3203372

[13] Ito HT, Schuman EM. Functional division of hippocampal area CA1 via modulatory gating of entorhinal cortical inputs. Hippocampus. 2012;22(2): 372–387. 10.1002/hipo.20909 21240920 PMC3627339

[14] Li XG, Somogyi P, Ylinen A, Buzsáki G. The hippocampal CA3 network: an in vivo intracellular labeling study. J Comp Neurol. 1994;339(2): 181–208. 10.1002/cne.903390204 8300905

[15] Ishizuka N, Cowan WM, Amaral DG. A quantitative analysis of the dendritic organization of pyramidal cells in the rat hippocampus. J Comp Neurol. 1995;362(1): 17–45. 10.1002/cne.903620103 8576427

[16] Amaral DG, Witter MP. The three-dimensional organization of the hippocampal formation: a review of anatomical data. Neuroscience. 1989;31(3): 571–591. 2687721 10.1016/0306-4522(89)90424-7

[17] Witter MP, Van Hoesen GW, Amaral DG. Topographical organization of the entorhinal projection to the dentate gyrus of the monkey. J Neurosci. 1989;9(1): 216–228. 2913203 10.1523/JNEUROSCI.09-01-00216.1989 PMC6570017

[18] Ishizuka N, Weber J, Amaral DG. Organization of intrahippocampal projections originating from CA3 pyramidal cells in the rat. J Comp Neurol. 1990;295(4): 580–623. 10.1002/cne.902950407 2358523

[19] Lu L, Leutgeb JK, Tsao A, Henriksen EJ, Leutgeb S, Barnes CA, et al Impaired hippocampal rate coding after lesions of the lateral entorhinal cortex. Nat Neurosci. 2013;16(8): 1085–1093. 10.1038/nn.3462 23852116

[20] Claiborne BJ, Amaral DG, Cowan WM. A light and electron microscopic analysis of the mossy fibers of the rat dentate gyrus. J Comp Neurol. 1986;246(4): 435–458. 10.1002/cne.902460403 3700723

[21] Witter MP. Intrinsic and extrinsic wiring of CA3: indications for connectional heterogeneity. Learn Mem. 2007;14(11): 705–713. 10.1101/lm.725207 18007015

[22] Chawla MK, Guzowski JF, Ramirez-Amaya V, Lipa P, Hoffman KL, Marriott LK, et al Sparse, environmentally selective expression of Arc RNA in the upper blade of the rodent fascia dentata by brief spatial experience. Hippocampus. 2005;15(5): 579–586. 10.1002/hipo.20091 15920719

[23] Raber J, Allen AR, Rosi S, Sharma S, Dayger C, Davis MJ, et al Effects of whole body (56) Fe radiation on contextual freezing and Arc-positive cells in the dentate gyrus. Behav Brain Res. 2013;246: 162–167. 10.1016/j.bbr.2013.02.022 23454674

[24] Nakamura NH, Flasbeck V, Maingret N, Kitsukawa T, and Sauvage MM. Proximodistal segregation of nonspatial information in CA3: Preferential recruitment of a proximal CA3-distal CA1 network in non-spatial recognition memory. J Neurosci. 2013;33(28): 11506–11514. 10.1523/JNEUROSCI.4480-12.2013 23843521 PMC6618684

[25] Flasbeck V, Atucha E, Nakamura NH, Yoshida M. and Sauvage MM. Spatial information is preferentially processed by the distal part of CA3: implication for memory retrieval. Behav Brain Res. 2018; 347: 116–123. 10.1016/j.bbr.2018.02.046 29518437

[26] Laurent F, Brotons-Mas JR, Cid E, Lopez-Pigozzi D, Valero M, Gal B. et. al Proximodistal Structure of Theta Coordination in the Dorsal Hippocampus of Epileptic Rats. J Neurosci. 2015;35(11): 4760–4775. 10.1523/JNEUROSCI.4297-14.2015 25788692 PMC6605134

[27] Hartzell AL, Burke SN, Hoang LT, Lister JP, Rodriguez CN, Barnes CA. Transcription of the immediate-early gene Arc in CA1 of the hippocampus reveals activity differences along the proximodistal axis that are attenuated by advanced age. J Neurosci. 2013;33(8): 3424–3433. 10.1523/JNEUROSCI.4727-12.2013 23426670 PMC3711759

[28] Lu L, Igarashi KM, Witter MP, Moser El, Moser MB. Topography of Place Maps along the CA3-to-CA2 Axis of the Hippocampus. Neuron. 2015;87(5): 1078–1092. 10.1016/j.neuron.2015.07.007 26298277

[29] Lee H, Wang C, Deshmukh SS, Knierim JJ. Neural Population Evidence of Functional Heterogeneity along the CA3 Transverse Axis: Pattern Completion versus Pattern Separation. Neuron. 2015;87(5): 1093–1105. 10.1016/j.neuron.2015.07.012 26298276 PMC4548827

[30] Kesner RP. Behavioral functions of the CA3 subregion of the hippocampus. Learn Mem. 2007;14(11): 771–781. 10.1101/lm.688207 18007020

[31] Farovik A, Dupont LM, Eichenbaum H. Distinct roles for dorsal CA3 and CA1 in memory for sequential non-spatial events. Learn Mem. 2009;17(1): 12–17. 10.1101/lm.1616209 20028733 PMC2807176

[32] Kraus BJ, Robinson RJ, White JA, Eichenbaum H, Hasselmo ME. Hippocampal “Time Cells”: Time versus Path Integration. Neuron.2013;78(6): 1090–1101. 10.1016/j.neuron.2013.04.015 23707613 PMC3913731

[33] Salz DM, Tiganj Z, Khasnabish S, Kohley A, Sheehan D, Howard MW, et al Time Cells in Hippocampal Area CA3. J Neurosci. 2016;36(28): 7476–84. 10.1523/JNEUROSCI.0087-16.2016 27413157 PMC4945667

[34] Cai DJ, Aharoni D, Shuman T, Shobe J, Biane J, Song W, et al A shared neural ensemble links distinct contextual memories encoded close in time. Nature. 2016;534(7605): 115–8. 10.1038/nature17955 27251287 PMC5063500

[35] MacDonald C, Lepage KQ, Eden UT, Eichenbaum H. Hippocampal “time cells” bridge the gap in memory for discontiguous events. Neuron. 2011;71(4): 737–749. 10.1016/j.neuron.2011.07.012 21867888 PMC3163062

[36] MacDonald C, Carrow S, Place R, Eichenbaum H. Distinct Hippocampal Time Cell Sequences Represent Odor Memories in Immobilized Rats. Neurosci. 2013;33(36): 14607–14616. 10.1523/JNEUROSCI.1537-13.2013 PMC3761059 24005311

[37] Sellami A, Al Abed AS, Brayda-Bruno L, Etchamendy N, Valério S, Oulé M, et al Temporal binding function of dorsal CA1 is critical for declarative memory formation. Proc Natl Acad Sci U S A. 2017;114(38): 10262–10267. 10.1073/pnas.1619657114 28874586 PMC5617244

[38] Mankin EA, Sparks FT, Slayyeh B, Sutherland RJ, Leutgeb S, Leutgeb JK. Neuronal code for extended time in the hippocampus. Proc Natl Acad Sci U S A. 2012;109(47): 19462–19467. 10.1073/pnas.1214107109 23132944 PMC3511087

[39] Dere E, Huston JP, De Souza Silva MA. Episodic-like memory in mice: simultaneous assessment of object, place and temporal order memory. Brain Res Protoc. 2005;16(1): 10–19. 10.1016/j.brainresprot.2005.08.001 16185914

[40] DeVito LM, Konigsberg R, Lykken C, Sauvage M, Young WS, Eichenbaum HJ. Vasopressin 1b receptor knock-out impairs memory for temporal order. J Neurosci. 2009;29(9): 2676–2683. Erratum in: J Neurosci. 29(15): 5044. 10.1523/JNEUROSCI.5488-08.2009 19261862 PMC2671073

[41] Place R, Lykken C, Beer Z, Suh J, McHugh T, Tonegawa S, et al NMDA signaling in CA1 mediates selectively the spatial component of episodic memory. Learn. Mem. 2012;19(4): 164–169. 10.1101/lm.025254.111 22419815 PMC3312619

[42] Inostroza M, Brotons-Mas JR, Laurent F, Cid E, de la Prida LM. Specific impairment of "what-where-when" episodic-like memory in experimental models of temporal lobe epilepsy. J Neurosci. 2013;33(45): 17749–17762. 10.1523/JNEUROSCI.0957-13.2013 24198366 PMC6618429

[43] Vazdarjanova A, Guzowski JF. Differences in hippocampal neuronal population responses to modifications of an environmental context: evidence for distinct, yet complementary, functions of CA3 and CA1 ensembles. J Neurosci. 2004;24(29): 6489–6496. 10.1523/JNEUROSCI.0350-04.2004 15269259 PMC6729865

[44] Paxinos G, Watson C. The rat brain in stereotaxic coordinates Academic press, San Diego 2007.

[45] Guzowski JF, Timlin JA, Roysam B, McNaughton BL, Worley PF, Barnes CA. Mapping behaviorally relevant neural circuits with immediate-early gene expression. Curr Opin Neurobiol. 2005;15(5): 599–606. 10.1016/j.conb.2005.08.018 16150584

[46] Kubik S, Miyashita T, Guzowski JF. Using immediate-early genes to map hippocampal subregional functions. Learn Mem. 2007;14(11): 758–770. 10.1101/lm.698107 18007019

[47] Sauvage MM, Nakamura NH, Beer Z. Mapping memory function in the medial temporal lobe with the immediate-early gene Arc. Behav Brain Res. 2013;254: 22–33. 10.1016/j.bbr.2013.04.048 23648768

[48] Shepherd JD, Bear MF. New views of Arc, a master regulator of synaptic plasticity. Nat Neurosci. 2011;14(3): 279–284. 10.1038/nn.2708 21278731 PMC8040377

[49] Guzowski JF, McNaughton BL, Barnes CA, Worley PF. Environment-specific expression of the immediate-early gene Arc in hippocampal neuronal ensembles. Nat Neurosci. 1999;2(12): 1120–1124. 10.1038/16046 10570490

[50] Guzowski JF, Setlow B, Wagner EK, McGaugh JL. Experience-dependent gene expression in the rat hippocampus after spatial learning: a comparison of the immediate-early genes Arc, c-fos, and zif268. J Neurosci. 2001;21(14): 5089–5098. 11438584 10.1523/JNEUROSCI.21-14-05089.2001 PMC6762831

[51] Ennaceur A, Delacour J. A new one-trial test for neurobiological studies of memory in rats. 1: Behavioral data. Behav Brain Res. 1988;31(1): 47–59. 3228475 10.1016/0166-4328(88)90157-x

[52] Vazdarjanova A, McNaughton B, Barnes C, Worley P, Guzowski JF. Experience-dependent coincident expression of the effector immediate-early genes arc and Homer 1a in hippocampal and neocortical neuronal networks. J Neurosci. 2002;22(23): 10067–10071. 12451105 10.1523/JNEUROSCI.22-23-10067.2002 PMC6758761

[53] Beer Z, Chwiesko C, Kitsukawa T, Sauvage MM. Spatial and stimulus-type tuning in the LEC, MEC, POR, PrC, CA1, and CA3 during spontaneous item recognition memory. Hippocampus. 2013;23(12): 1425–1438. 10.1002/hipo.22195 23966131

[54] Beer Z, Chwiesko C, Sauvage MM. Processing of spatial and non-spatial information reveals functional homogeneity along the dorso-ventral axis of CA3, but not CA1. Neurobiol Learn Mem. 2014;111: 56–64. 10.1016/j.nlm.2014.03.001 24657342

[55] West MJ. Stereological methods for estimating the total number of neurons and synapses: issues of precision and bias. Trends Neurosci. 1999;22(2): 51–61. 10092043 10.1016/s0166-2236(98)01362-9

[56] West MJ. New stereological methods for counting neurons. Neurobiol Aging. 1993;14(4): 275–85. 8367009 10.1016/0197-4580(93)90112-o

[57] Vazdarjanova A, Ramirez-Amaya V, Insel N, Plummer TK, Rosi S, Chowdhury S, et al Spatial exploration induces ARC, a plasticity-related immediate-early gene, only in calcium/calmodulin-dependent protein kinase II-positive principal excitatory and inhibitory neurons of the rat forebrain. J Comp Neurol. 2006;498(3): 317–29. 10.1002/cne.21003 16871537

[58] Gelman A, Hill J. Data analysis using regression and multilevel/hierarchical models Cambridge University Press New York, NY, USA 2007.

[59] Singmann H, Bolker B, Westfall J, Aust F. afex: Analysis of Factorial Experiments. Available from: https://cran.r-project.org/package=afex. [cited 2016 July 25].

[60] Bates D, Maechler M, Bolker B, Walker S. lme4: Linear mixed-effects models using Eigen and S4. R package version 1.1–9, https://CRAN.R-project.org/package=lme4. Available from: https://cran.r-project.org/package=lme4. [cited 2017 September 25].

[61] Lenth RV. Least-Squares Means: The R Package Ismeans. Journal of Statistical Software, 69(1). https://doi.org/10.18637/jss.v069.i01. 2016.

[62] Barr DJ, Levy R, Scheepers C, Tily H J. Random effects structure for confirmatory hypothesis testing: Keep it maximal. Journal of Memory and Language. 2013;68(3): 255–278. 10.1016/j.jml.2012.11.001 PMC3881361 24403724

[63] Kesner RP, Hunsaker MR, Gilbert PE. The role of CA1 in the acquisition of an object-trace-odor paired associate task. Behav Neurosci. 2005;119(3): 781–786. 10.1037/0735-7044.119.3.781 15998199

[64] Itskov V, Curto C, Pastalkova E, Buzsáki G. Cell assembly sequences arising from spike threshold adaptation keep track of time in the hippocampus. J Neurosci. 2011;31(8): 2828–2834. 10.1523/JNEUROSCI.3773-10.2011 21414904 PMC3097063

[65] Morrissey MD, Maal-Bared G, Brady S, Takehara-Nishiuchi K. Functional dissociation within the entorhinal cortex for memory retrieval of an association between temporally discontiguous stimuli. J Neurosci. 2012;32(16): 5356–5361. 10.1523/JNEUROSCI.5227-11.2012 22514300 PMC6703475

[66] Barker GR, Bird F, Alexander V, Warburton EC. Recognition memory for objects, place, and temporal order: a disconnection analysis of the role of the medial prefrontal cortex and perirhinal cortex. J Neurosci. 2007;27(11): 2948–2957. 10.1523/JNEUROSCI.5289-06.2007 17360918 PMC6672574

[67] Naya Y, Suzuki WA. Integrating what and when across the primate medial temporal lobe. Science. 2011;333(6043): 773–776. 10.1126/science.1206773 21817056

[68] Olarte-Sánchez CM, Kinnavane L, Amin E, Aggleton JP. Contrasting networks for recognition memory and recency memory revealed by immediate-early gene imaging in the rat. Behav Neurosci. 2014;128(4): 504–522. 10.1037/a0037055 24933661 PMC4105319

[69] Atsuo S, Sugar J, Lu L. Wang C, Knierium JJ, Moser MB, Moser EI. Integrating time in lateral entorhinal cortex. Abstract in Society for Neuroscience 2017. Washington, DC. Poster nr: 084.21/SS20.

[70] Ku SP, Nakamura NH, Maingret N, Mahnke L, Yoshida M, Sauvage MM. Regional specific evidence for memory-load dependent activity in the dorsal subiculum and the lateral entorhinal cortex. Front Syst Neurosci. 2017;11: 51 10.3389/fnsys.2017.00051 28790897 PMC5524887

[71] Save E, Sargolini F. Disentangling the role of the MEC and LEC in the processing of spatial and non-spatial information: contribution of lesion studies. Front Syst Neurosci. 2017;11: 81 10.3389/fnsys.2017.00081 29163076 PMC5663729

[72] Robinson NTM, Priestley JB, Rueckemann JW, Garcia AD, Smeglin VA, Marino FA et al Medial Entorhinal Cortex Selectively Supports Temporal Coding by Hippocampal Neurons. Neuron. 2017;94(3): 677–688. 10.1016/j.neuron.2017.04.003 28434800 PMC5465388

[73] Schlesiger MI, Cannova CC, Boublil BL, Hales JB, Mankin EA, Brandon MP et al The medial entorhinal cortex is necessary for temporal organization of hippocampal neuronal activity. Nat Neurosci. 2015;18(8): 1123–1132. 10.1038/nn.4056 26120964 PMC4711275

[74] Kraus BJ, Brandon MP, Robinson RJ, Connerney MA, Hasselmo ME, Eichenbaum H. During Running in Place, Grid Cells Integrate Elapsed Time and Distance Run. Neuron. 2015;88(3): 578–589. 10.1016/j.neuron.2015.09.031 26539893 PMC4635558

[75] Rolls E. Spatial view cells and the representation of place in the primate hippocampus. Hippocampus. 1999;9(4): 467–480. 10.1002/(SICI)1098-1063(1999)9:4<467::AID-HIPO13>3.0.CO;2-F 10495028

[76] Nakazawa K, McHugh T, Wilson M, Tonegawa S. NMDA receptors, place cells and hippocampal spatial memory. Nat Rev Neurosci. 2004;5(5): 361–372. 10.1038/nrn1385 15100719

[77] Van Cauter T, Camon J, Alvernhe A, Elduayen C, Sargolini F, Save E. Distinct roles of medial and lateral entorhinal cortex in spatial cognition. Cereb Cortex. 2013;23(2): 451–459. 10.1093/cercor/bhs033 22357665

[78] Wilson DI, Langston RF, Schlesiger MI, Wagner M, Watanabe S, Ainge JA. Lateral entorhinal cortex is critical for novel object-context recognition. Hippocampus. 2013;23(5): 352–366. 10.1002/hipo.22095 23389958 PMC3648979

[79] Tsao A, Moser MB, Moser EI. Traces of experience in the lateral entorhinal cortex. Curr Biol. 2013;23(5): 399–405. 10.1016/j.cub.2013.01.036 23434282

[80] Kesner RP, Lee I, Gilbert P. A behavioral assessment of hippocampal function based on a subregional analysis. Rev Neurosci. 2004;15(5): 333–351. 15575490 10.1515/revneuro.2004.15.5.333

[81] Rolls ET, Kesner RP. A computational theory of hippocampal function, and empirical tests of the theory. Prog Neurobiol. 2006;79(1): 1–48. 10.1016/j.pneurobio.2006.04.005 16781044

[82] Gilbert PE, Kesner RP. Localization of function within the dorsal hippocampus: the role of the CA3 subregion in paired-associate learning. Behav Neurosci. 2003;117(6): 1385–1394. 10.1037/0735-7044.117.6.1385 14674856

